# The Impact of Cholecystectomy on the Gut Microbiota: A Case-Control Study

**DOI:** 10.3390/jcm8010079

**Published:** 2019-01-11

**Authors:** Won Jae Yoon, Han-Na Kim, Eunkyo Park, Seungho Ryu, Yoosoo Chang, Hocheol Shin, Hyung-Lae Kim, Sun Young Yi

**Affiliations:** 1 Department of Internal Medicine, College of Medicine, Ewha Womans University, Seoul 07985, Korea; biliary@naver.com; 2 Medical Research Institute, Kangbuk Samsung Hospital, Sungkyunkwan University School of Medicine, Seoul 03181, Korea; hanna147942@gmail.com; 3 Department of Biochemistry, College of Medicine, Ewha Womans University, Seoul 07985, Korea; eunkyo85@gmail.com; 4 Center for Cohort Studies, Total Healthcare Center, Kangbuk Samsung Hospital, Sungkyunkwan University School of Medicine, Seoul 03181, Korea; sh703.yoo@samsung.com (S.R.); yoosoo.chang@samsung.com (Y.C.); 5 Department of Occupational and Environmental Medicine, Kangbuk Samsung Hospital, Sungkyunkwan University School of Medicine, Seoul 03181, Korea; 6 Department of Family Medicine, Kangbuk Samsung Hospital, Sungkyunkwan University School of Medicine, Seoul 03181, Korea; hcfm.shin@samsung.com

**Keywords:** cholecystectomy, microbiota, gastrointestinal microbiome, 16S rRNA

## Abstract

Cholecystectomy alters the bile flow into the intestine and the enterohepatic circulation of the bile acids; this may affect the gut microbiota. We assessed the gut microbiota composition of patients who had undergone cholecystectomy and compared with those who had not. From a cohort of 1463 adult participants who underwent comprehensive health screening examinations, 27 subjects who had undergone cholecystectomy (cholecystectomy group) and 81 age- and sex-matched subjects who had not (control group) were selected. Clinical parameters were collected and compared. Microbial composition was determined by 16S rRNA gene sequencing of DNA extracted from fecal samples. We evaluated differences in the overall microbial composition and in the abundance of taxa. The two groups were comparable with respect to clinical characteristics and laboratory results. The actual number of taxa observed in a sample (observed features) was significantly lower in the cholecystectomy group than in the control group (*p* = 0.042). The beta diversity of Jaccard distance index was significantly different between the two groups (*p* = 0.027). *Blautia obeum* and *Veillonella parvula* were more abundant in the cholecystectomy group. The difference in the diversity of the gut microbiota between the cholecystectomy and control groups was subtle. However, *B. obeum* and *V. parvula*, which have azoreductase activity, were more abundant in the cholecystectomy group. The impact of such changes in the gut microbiota on health remains to be determined.

## 1. Introduction

The gallbladder (GB) is an organ of the digestive system that stores and concentrates bile between meals. The GB contracts and releases bile into the small intestine in response to feeding. Bile acids entering the intestines facilitate the absorption of dietary lipids. The GB influences bile flow into the intestine and enterohepatic circulation of bile acids. The GB, with its absorptive and secretory functions, thus contributes to the composition of the bile flowing into the intestine [1].

Cholecystectomy, the surgical removal of the GB, is one of the most commonly performed surgical procedures. It was the eighth most common surgical procedure in the United States in 2011 [2]. Symptomatic gallstones, cholecystitis, and GB tumors are indications for cholecystectomy [3]. Cholecystectomy generally decreases the size of bile acid pool and increases the enterohepatic recirculation rates of bile acids [1]. It also increases the exposure of the bile acid pool to intestinal bacteria, resulting in increased bacterial deconjugation and dihydroxylation of bile acids, resulting in an increase in the proportion of secondary bile acids [4,5,6,7]. A previous study demonstrated the presence of the ketohydroxy bile acids in cholecystectomized patients; the authors suggested the possibility of an altered gut microbiota [4]. Some have advocated that cholecystectomy may increase the risk of colorectal cancer (CRC) [8,9,10,11,12,13,14,15].

Gut microbiota dysbiosis is associated with cholesterol gallstones [16,17]. There have been a number of reports linking gut microbiota with the development of CRC [18]. A study comparing the changes in fecal microbiota of gallstone patients before and after cholecystectomy reported that the phylum Bacteroidetes was increased post-cholecystectomy [19]. However, there are few studies comparing fecal microbiota of subjects who had and had not undergone cholecystectomy. We hypothesized that the fecal microbiota would differ depending on receiving cholecystectomy or not. We assessed the fecal microbiota composition by 16S rRNA gene sequencing.

## 2. Materials and Methods

### 2.1. Subjects

Participants were recruited from men and women of the Kangbuk Samsung Cohort Study of Korea who undergo comprehensive annual or biennial examinations at the Kangbuk Samsung Hospital Healthcare Screening Center in the Republic of Korea. Stool samples were collected from 1463 adult participants who had undergone comprehensive health-screening examinations between June and September 2014.

From this cohort, 27 subjects who had undergone cholecystectomy (cholecystectomy group) and 81 subjects who had not (control group) were selected; the subjects were matched for age (within 3 years) and sex. No subject had used antibiotics within 6 weeks prior to enrollment, or cholesterol-lowering medications or probiotics within 4 weeks prior to enrollment. The clinical parameters age, sex, body mass index (BMI), history of smoking, alcohol intake, dietary intake, and laboratory results were collected and compared.

Ethical approval for the phenotype, genotype, and microbiota studies within the Kangbuk Samsung Cohort Study was provided by the Institutional Review Boards of Kangbook Samsung Hospital (KBSMC 2013-01-245-12) and Ewha Womans University Mokdong Hospital (EUMC 2017-08-037-001). Written consent was obtained from all participants after the nature and possible consequences of the studies were explained in detail. All applicable institutional and governmental regulations concerning the ethical use of human volunteers were observed during this research. The research was carried out in compliance with the Declaration of Helsinki.

### 2.2. Fecal Samples and DNA Extraction

Fecal samples were frozen immediately after defecation at −20 °C and were placed at −70 °C within 24 h. Within 1 month, DNA was extracted from the fecal samples using the PowerSoil^®^ DNA Isolation Kit (MO BIO Laboratories, Carlsbad, CA, USA) according to the manufacturer’s instructions.

### 2.3. PCR Amplification and Sequencing of the Bacterial 16S rRNA Gene

The variable V3 and V4 regions of the 16S rRNA gene were amplified using the universal primers 341F (5′-TCG TCG GCA GCG TCA GAT GTG TAT AAG AGA CAG CCT ACG GGN GGC WGC AG-3′) and 805R (5′-GTC TCG TGG GCT CGG AGA TGT GTA TAA GAG ACA GGA CTA CHV GGG TAT CTA ATC C-3′), with each primer modified to contain a unique 8-nucleotide barcode index using the Nextera XT DNA Library Preparation kit (Illumina, San Diego, CA, USA). Each PCR contained 5 ng/µL DNA template, 2× KAPA HiFi HotStart Ready Mix (KAPA Biosystems, Wilmington, MA, USA), and 2 pmol of each primer. Reaction conditions consisted of an initial incubation at 95 °C for 3 min, followed by 25 cycles of 95 °C for 30 s, 55 °C for 30 s, and 72 °C for 30 s. The samples were subjected to a final extension at 72 °C for 5 min. After PCR clean-up and index PCR, sequencing was performed on the Illumina MiSeq platform following the manufacturer’s instructions [20,21].

### 2.4. 16S rRNA Gene Compositional Analysis

DADA2 pipeline [22] within the QIIME2 package (version 2017.12, https://qiime2.org) [23] was used to filter low-quality and chimeric sequences and to generate unique amplicon sequence variants (ASVs). Since unique grouping sequences produce the operational taxonomic units (OTUs) from DADA2, they are regarded as 100% of the OTU and are referred to as sequence variants. QIIME2 was used to construct the FeatureTable, which is the equivalent of the biom table, and the representative sequence files. The sequencing depth ranged from 2528 to 51,419 reads per sample (mean = 23,115, standard deviation = 12,722) with 10,612 features. The sequences were mapped at 99% sequence identity to an optimized version of the GreenGenes database (version 13.8) containing the V3–V4 region to determine taxonomies.

### 2.5. Statistical Analysis

Basic statistical analyses were performed using Stata 12.1 (StataCorp LP, College Station, TX, USA). For categorical data, the chi-squared test or Fisher’s exact test was performed, as appropriate. The Wilcoxon rank–sum test was used for comparison of the quantitative clinical variables. A two-sided *p*-value < 0.05 was considered statistically significant. Exploratory and differential microbial composition analyses were conducted in QIIME2 [23]. The actual number of taxa observed in a sample (“observed”) and Shannon’s diversity index, which is a measure of diversity based on both richness and evenness [24], were used. Additionally, alpha diversity was measured using an indicator of phylogenetic diversity (PD), the Faith’s PD, often referred to as PD [25]. The Kruskal–Wallis test was used to estimate the median of the difference between the cholecystectomy and control groups.

To compare beta diversity, phylogenetic methods including the unweighted and weighted UniFrac distances [26] were used for the present/absolute and abundance data, respectively. Additionally, non-phylogenetic methods were used with the Jaccard and Bray–Curtis distances [27] for the present/absolute and abundance data, respectively. The tests of significance were performed using permutational multivariate analysis of variance (PERMANOVA) with 999 permutations.

Relationships between the abundance of one or more taxa and cholecystectomy were examined from the phylum to species levels. A multivariate analysis of cholecystectomy and bacterial data was performed using MaAsLin (https://huttenhower.sph.harvard.edu/maaslin) [28] in RStudio (version 0.98.983) and the Benjamini–Hochberg method to adjust for multiple testing. All analyses in MaAsLin were performed using the default options. We used the zero-inflated regression model on a Gaussian distribution for zero-inflated microbiota data. The resulting *p*-values were corrected for multiple comparisons at each phylogenetic level and each personality trait by Benjamini–Hochberg correction. A *q*-value < 0.05 was considered indicative of statistical significance. As the case and control groups were matched for age and sex, no adjustment was performed.

## 3. Results

### 3.1. Demographic Characteristics

A total of 108 subjects (27 in the cholecystectomy group and 81 in the control group) were analyzed. The two groups were comparable with respect to the clinical characteristics of age, sex, BMI, history of smoking, alcohol intake, and dietary intake (Table 1). The laboratory results of the two groups were not significantly different, with the exception of the median serum alanine transaminase level, which was significantly higher in the cholecystectomy group, albeit within the normal limits (Table 2).

### 3.2. Gut Microbial Diversity

**Figure 1:**
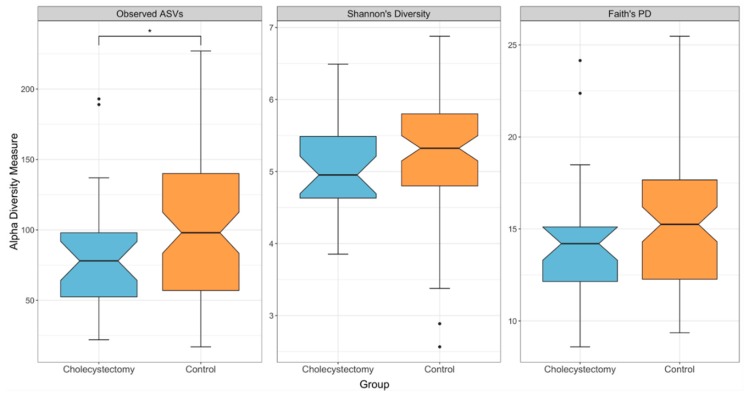
Boxplots of the alpha-diversity indices in the cholecystectomy and control groups. From left to right, observed amplicon sequence variants (ASVs), Shannon’s diversity index, and Faith’s phylogenetic diversity (cholecystectomy group, *n* = 27; control group, *n* = 81). * *q* < 0.05 (Kruskal–Wallis test, Benjamini–Hochberg correction).

The actual number of taxa observed in a sample (“observed”) was significantly lower in the cholecystectomy group (*p* = 0.042, *H* = 4.118, Kruskal–Wallis test) (Figure 1). However, there were no significant differences in the other two alpha diversity measures, although trends similar to that of the “observed” index were found (Shannon’s diversity index: *p* = 0.264, *H* = 1.249; Faith’s PD: *p* = 0.170, *H* = 1.885, Kruskal–Wallis test).

**Figure 2:**
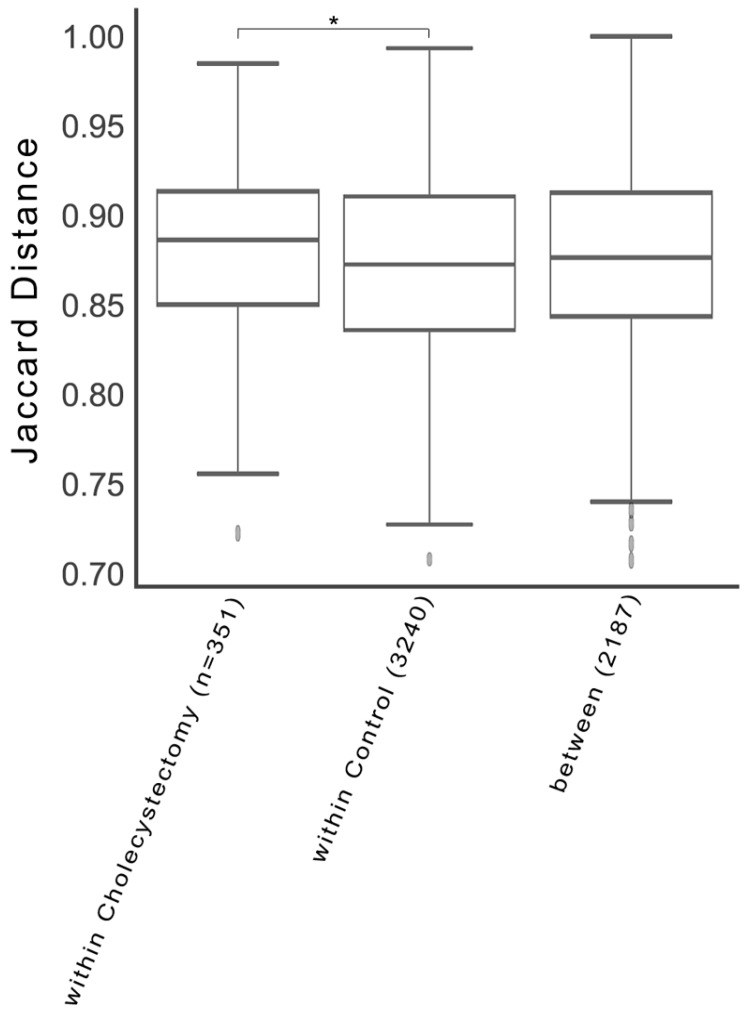
Beta-diversity indices of the cholecystectomy and control groups. From left, the Jaccard distances within the cholecystectomy group, within the control group, and between the cholecystectomy and control groups. * *q* = 0.027 (pseudo-*F* = 1.213, permutations = 999, PERMANOVA).

Regarding the Jaccard distance, the gut microbiota of the cholecystectomy group exhibited a higher level of dissimilarity than that of the control group (*p* = 0.027, pseudo-*F* = 1.21, pair-wise PERMANOVA) (Figure 2). However, the Bray–Curtis distance (*p* = 0.449, pseudo-*F* = 1.003, PERMANOVA), unweighted UniFrac distance (*p* = 0.205, pseudo-*F* = 1.215, PERMANOVA), and weighted UniFrac distance (*p* = 0.780, pseudo-*F* = 0.503, PERMANOVA) were not significantly different. Comparison of the bacterial communities by principal coordinate analysis using the beta diversity indices showed no differences between the two groups (Supplementary Figure S1).

### 3.3. Gut Microbiota Composition

**Figure 3:**
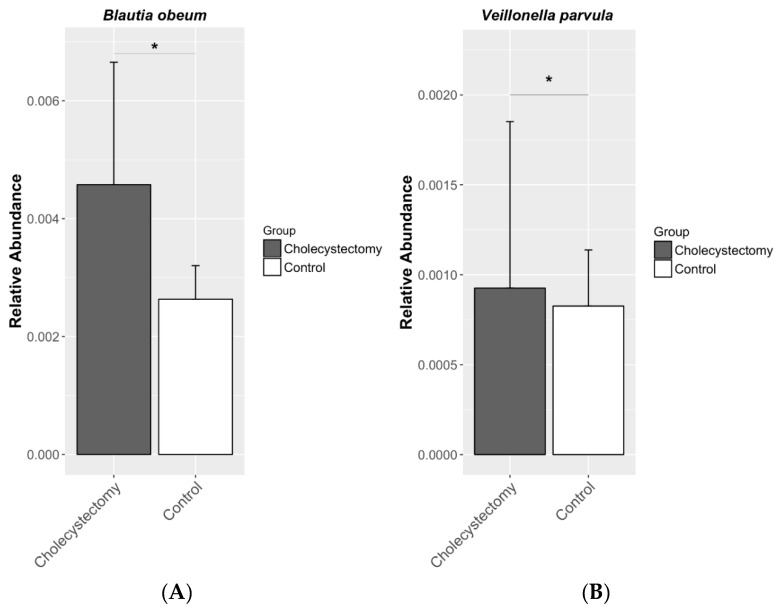
Relative abundances of (**A**) *Blautia obeum* and (**B**) *Veillonella parvula* in the gut microbiota. Both species were more abundant in the cholecystectomy group. * *q* < 0.05 (generalized linear models, Benjamini–Hochberg correction).

Table 3 shows the seven candidate taxa correlated with cholecystectomy when a false discovery rate of *q* < 0.25 (the threshold employed in previous microbiome studies that allows compensation for multiple microbial taxa and comparison adjustments [19]) was applied. *Blautia obeum* and *Veillonella parvula* belonging to the phylum Firmicutes were more abundant in the cholecystectomy group (coefficient = 0.457, *q* = 0.024; coefficient = 0.980, *q* = 0.044, respectively). The abundance of family S24_7 belonging to the phylum Bacteroidetes was decreased (coefficient = −0.558, *q* = 0.061), and that of the family Lactobacillaceae belonging to the phylum Firmicutes was increased in the cholecystectomy group (coefficient = 0.504, *q* = 0.061) (Figure 3). At the genus level, the abundance of *Ruminococcus* was greater in the cholecystectomy group (coefficient = 0.334, *q* = 0.210).

## 4. Discussion

We report here that the gut microbiota composition differs between subjects who underwent cholecystectomy and those who did not. The actual number of taxa observed in a sample (“observed”) was significantly lower in the cholecystectomy group. The species *B. obeum* and *V. parvula*, members of the phylum Firmicutes, were more abundant in the cholecystectomy group. To our knowledge, this study is one of the first to compare the gut microbiota between subjects who underwent cholecystectomy and those who did not.

Cholecystectomy increases the enterohepatic recirculation rates of bile acids and increases the exposure of the bile acid pool to intestinal bacteria [1,4,5,6,7]. A study reported in the early 1970s predicted an alteration of the gut microbiota in cholecystectomized patients [4]. One prior study compared the fecal microbiota composition of patients with gallstones before and after cholecystectomy. Compared with control subjects, patients with gallstones had a higher overall fecal bile acid concentrations. Before cholecystectomy, the abundances of the genus *Roseburia* and the species *Bacteroides uniformis* were decreased, and those of the family Ruminococcaceae and the genus *Oscillospira* were increased in patients with gallstones compared with the controls. In the patients with gallstones, the abundance of the phylum Bacteroidetes showed a significant increase after cholecystectomy [19]. Another study which compared fecal microbiota of cholecystectomy patients with that of a healthy population demonstrated higher abundances of genera *Bifidobacterium*, *Anaerostipes*, and *Dorea* in cholecystectomy patients [29].

In our study, *B. obeum* and *V. parvula*, members of the phylum Firmicutes, were more abundant in the cholecystectomy group. *B. obeum* has been reclassified from *Ruminococcus obeum* to *B. obeum* [30]. In a previous study, the abundance of members of the family Ruminococcaceae was positively correlated with the levels of secondary bile acids in the intestine [31]. The genus *Ruminococcus* showed a trend of being more abundant in the cholecystectomy group. *Ruminococcus* is currently considered a polyphyletic genus, with species members belonging to the Ruminococcaceae and Lachnospiraceae [32]. The abundance of *Ruminococcus* is reportedly increased in patients with gastric neoplasm and CRC [33,34].

*V. parvula* is a strictly anaerobe Gram-negative biofilm-forming commensal coccus found in the human mouth, lungs, vagina, and gastrointestinal tract [35,36,37]. It has an outer membrane comprising lipopolysaccharides [35]. It may be an opportunistic pathogen and has been reported to be involved in endocarditis [38,39], urinary tract infections [40], epidural abscess [41], meningitis [42,43], bacteremia [44], osteomyelitis [45], and discitis [46]. Interestingly, a comparison of the fecal microbiota of patients with irritable bowel syndrome (IBS) with healthy controls showed that patients with constipation-predominant IBS had an increased abundance of *Veillonella* species [47].

The impact of changes in the gut microbiota after cholecystectomy on health remains to be determined. Cholecystectomy is known to be associated with an increased risk of CRC, especially right-sided colon cancer [8,9,10,11,12,13,14,15]. A recent meta-analysis of 10 cohort studies reported that cholecystectomy was associated with an increased risk of CRC [48]. The proposed mechanisms of the increased risk of CRC in patients who underwent cholecystectomy include continuous bile flow into the bowel [6] and increased concentrations of secondary bile acids in the bile [8], which is considered carcinogenic [49]. Thus, changes in the gut microbiota composition might play a role in the development of CRC. *B. obeum* and *V. parvula* exhibit azoreductase activity, which is linked to an increased risk of CRC [50].

This study has some limitations, including its retrospective and single-center nature. We could not evaluate whether the subjects had undergone endoscopic sphincterotomy, which may cause further alterations in bile flow. We were unable to determine the fecal bile acid concentrations or composition, which may affect the gut microbiota composition. This study included only people from the Korean population with a potentially similar diet and microbiota. In populations in other areas the world with different ethnicity and diet, the composition of microbiota may be different. Indeed, aforementioned study showed that the phylum Bacteroidetes was increased after cholecystectomy, which was not found in the present study [19]. However, this is one of the largest population-based studies to evaluate the gut microbiota of subjects who underwent cholecystectomy compared with those who did not. There were no significant differences in the clinical characteristics, including dietary composition, between the two groups; any such differences would likely have affected the gut microbiota composition [19].

## 5. Conclusions

In conclusion, there was little difference in the diversity of the gut microbiota between the cholecystectomy and control groups. However, there was a significant difference in the abundance of species possessing azoreductase activity. Whether such differences in the gut microbiota after cholecystectomy affect health remains to be determined.

## Figures and Tables

**Table 1:** Clinical characteristics of the subjects.

Characteristics	Cholecystectomy Group	Control Group	Missing, *n*	*p*-Value
Number (*n*)	27	81		
Age (years)	48 (39–64)	47 (38–64)		0.823
Sex (male:female)	15:12	45:36		1.000
BMI (kg/m^2^)	23.8 (19.4–30.2)	23.3 (18.7–29.5)		0.321
Smoking history (never:former:current)	15:7:2	50:17:10	7	0.778 *
Frequency of alcohol consumption (days/week)	1 (0–6)	1 (0–7)	10	0.543
Carbohydrate intake (g/day)	221.1 (24.6–477.5)	250.6 (0.9–474.9)	29	0.673
Fat intake (g/day)	19.5 (6.8–72.6)	23.5 (0–68.9)	29	0.653
Protein intake (g/day)	40.1 (12.9–108.5)	46.1 (0.3–107.3)	29	0.748

Values are numbers or medians (range); BMI, body mass index; * Fisher’s exact test.

**Table 2:** Laboratory parameters.

Characteristics	Cholecystectomy Group	Control Group	Missing, *n*	*p*-Value
Number (*n*)	27	81		
White blood cells (×10^3^/mm^3^)	5.5 (3.9–8.8)	5.7 (2.3–10.5)		0.273
Hemoglobin (g/dL)	13.8 (10.3–17.3)	14.2 (6.9–16.7)		0.823
Platelets (×10^3^/mm^3^)	234 (162–383)	238 (79–465)		0.859
Total cholesterol (mg/dL)	185 (144–269)	194 (126–275)		0.333
Triglycerides (mg/dL)	108 (32–256)	96 (34–432)		0.731
HDL-C (mg/dL)	55 (33–98)	56 (30–121)		0.887
LDL-C (mg/dL)	114 (85–199)	118.5 (60–194)	1	0.268
Total protein (g/dL)	7.2 (6.7–7.8)	7.1 (6.4–8.3)		0.751
Bilirubin (mg/dL)	0.9 (0.3–1.8)	0.8 (0.3–2.1)		0.516
AST (IU/L)	20 (13–39)	19 (12–32)		0.127
ALT (IU/L)	18 (9–65)	16 (7–40)		0.012
GGT (IU/L)	26 (6–180)	17.5 (6–81)	1	0.283
BUN (mg/dL)	13.8 (8.1–19.3)	14.1 (6.7–25.1)		0.717
Creatinine (mg/dL)	0.8 (0.6–1.2)	0.9 (0.5–1.2)		0.635
Glucose (mg/dL)	94 (76–136)	92 (75–130)		0.842
Hemoglobin A1c (%)	5.5 (5.1–7.5)	5.5 (5.1–6.8)		0.225

Values are numbers or medians (range); HDL-C, high-density lipoprotein-cholesterol; LDL-C, low-density lipoprotein-cholesterol; AST, aspartate transaminase; ALT, alanine transaminase; GGT, gamma-glutamyltransferase; BUN, blood urea nitrogen.

**Table 3:** Associations between cholecystectomy and gut microbiota composition using MaAsLin analysis.

Taxonomic Level	Taxa	CE ^¶^	Odds ^¶^	*n*	*n* not 0	*p*-Value	*q*-Value
Family	p_Bacteroidetes; c_Bacteroidia; o_Bacteroidales; f_S24_7	−0.558	0.572	108	42	0.004	0.061
Family	p_Firmicutes; c_Bacilli; o_Lactobacillales; f_Lactobacillaceae	0.504	1.655	108	33	0.004	0.061
Genus	p_Firmicutes; c_Clostridia; o_Clostridiales; f_Lachnospiraceae; g_Ruminococcus	0.344	1.411	108	89	0.005	0.210
Species	p_Firmicutes; c_Clostridia; o_Clostridiales; f_Lachnospiraceae; g_*Blautia*; s_*obeum*	0.457	1.579	108	41	0.001	0.025
Species	p_Firmicutes; c_Clostridia;o_Clostridiales; f_Veillonellaceae; g_*Veillonella*; s_*parvula*	0.980	2.664	108	13	0.003	0.044
Species	p_Actinobacteria; c_Actinobacteria; o_Bifidobacteriales; f_Bifidobacteriaceae; g_*Bifidobacterium*; s_*adolescentis*	0.476	1.610	108	44	0.009	0.074
Species	p_Firmicutes; c_Clostridia; o_Clostridiales; f_Lachnospiraceae; g_*Ruminococcus*; s_*torques*	0.477	1.611	108	41	0.009	0.074

CE, coefficient; odds, exponentiated CE; *n*, number of samples; p_, phylum; c_, class; o_, order; f_, family; g_, genus; s_, species; Results with a *q*-value < 0.25 are listed. *q*-values were calculated by Benjamini–Hochberg correction. ^¶^ Reference, control.

## Data Availability

All data generated or analyzed during this study are included in this published article and its Supplementary Information files. The raw 16S rRNA gene sequencing data sets (fastq files) and age and sex information are available in the Clinical and Omics Data Archive (http://coda.nih.go.kr/coda/frt/index.do, accession number: R000635) at the Korean National Institute of Health.

## Supplementary Materials

The following are available online at http://www.mdpi.com/2077-0383/8/1/79/s1, Figure S1: Principal coordinate analysis based the Jaccard distance index in cholecystectomy and control groups.
