# Supplementary figures and images for: The Impact of Cholecystectomy on the Gut Microbiota: A Case-Control Study
Source: J Clin Med. 2019 Jan 11;8(1):79. doi: 10.3390/jcm8010079 (PMC6352247; doi:10.3390/jcm8010079)

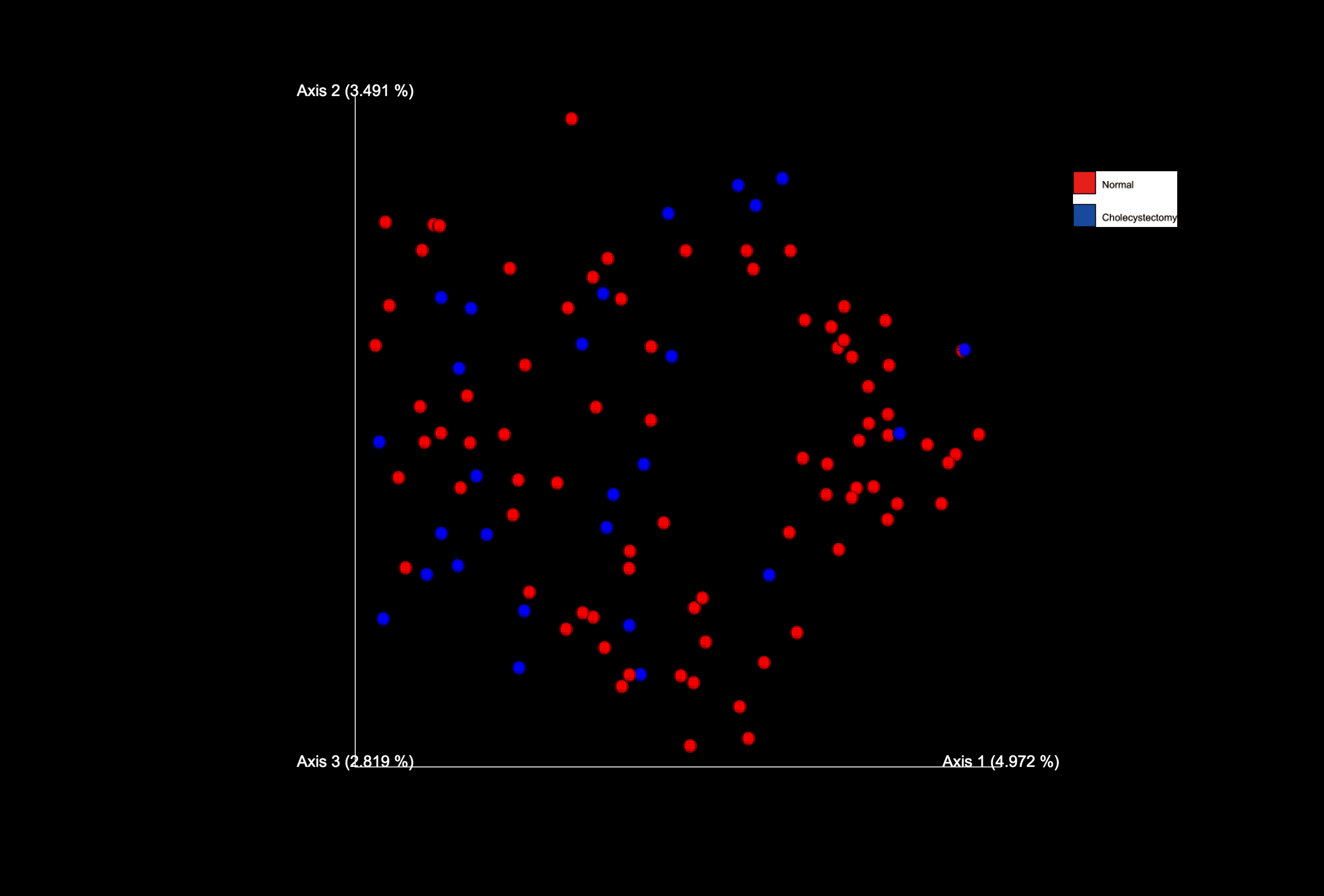

Supplement: Supplementary file 1 [file jcm-08-00079-s001.zip › jcm-425087.tif]
